# Improving the Performance of Machine Learning-Based Network Intrusion Detection Systems on the UNSW-NB15 Dataset

**DOI:** 10.1155/2021/5557577

**Published:** 2021-06-15

**Authors:** Soulaiman Moualla, Khaldoun Khorzom, Assef Jafar

**Affiliations:** Department of Telecommunication, Higher Institute for Applied Sciences and Technology, Damascus, Syria

## Abstract

Networks are exposed to an increasing number of cyberattacks due to their vulnerabilities. So, cybersecurity strives to make networks as safe as possible, by introducing defense systems to detect any suspicious activities. However, firewalls and classical intrusion detection systems (IDSs) suffer from continuous updating of their defined databases to detect threats. The new directions of the IDSs aim to leverage the machine learning models to design more robust systems with higher detection rates and lower false alarm rates. This research presents a novel network IDS, which plays an important role in network security and faces the current cyberattacks on networks using the UNSW-NB15 dataset benchmark. Our proposed system is a dynamically scalable multiclass machine learning-based network IDS. It consists of several stages based on supervised machine learning. It starts with the Synthetic Minority Oversampling Technique (SMOTE) method to solve the imbalanced classes problem in the dataset and then selects the important features for each class existing in the dataset by the Gini Impurity criterion using the Extremely Randomized Trees Classifier (Extra Trees Classifier). After that, a pretrained extreme learning machine (ELM) model is responsible for detecting the attacks separately, “One-Versus-All” as a binary classifier for each of them. Finally, the ELM classifier outputs become the inputs to a fully connected layer in order to learn from all their combinations, followed by a logistic regression layer to make soft decisions for all classes. Results show that our proposed system performs better than related works in terms of accuracy, false alarm rate, Receiver Operating Characteristic (ROC), and Precision-Recall Curves (PRCs).

## 1. Introduction

Nowadays, the rapid evolution of IoT, cloud, and big data domains has now reached an indescribable level, and the urgent need to use them has become unavoidable.

The prevailing data through the emerging technologies have many steps in their life cycle including creation, transfer, storage, and deletion. The portable information in the data has great importance at any stage of its cycle, especially when it is related to financial transactions or governments or the military. Consequently, data privacy and information security were fundamental issues for reducing losses that occur by overlooking them [1].

Due to systems vulnerabilities, intruders try to steal or destroy or alter the information and often damage the systems themselves.

Thus, information security in terms of confidentiality, integrity, and availability (CIA triad) must be taken into consideration when developing systems.

IDS is one of the most common issues in the field of cybersecurity to meet the challenges of any malicious cyberattacks.

IDS is used to detect suspicious activities on the network, network-based IDS, or on the host, host-based IDS, or on both of them, hybrid IDS. It may be either software or hardware or a combination of both.

IDSs are divided into three groups based on the methodology: signature-based IDS matching the traffic flow with stored signatures of known attacks, specification-based IDS applying a set of rules in the incoming packets to monitor any skewness from the normal behavior, and anomaly-based IDS sniffing the suspicious threats [2].

With the proliferation of attacks, the signature-based types suffer from continuously updating their databases, and the specification-based types need more expert knowledge to capture the new undesired traffic.

Because detection of anomalies is considered a classification problem in the world of machine learning, the use of machine learning methods as classifiers for IDS has increased [3], which is known as machine learning-based IDS, a branch of anomaly-based IDS.

Many labs have created datasets to help planning machine learning-based IDSs. The UNSW-NB15 dataset draws much attention from cybersecurity researchers with the latest cyberattacks.

In order to reduce misclassification, SMOTE was proposed as a very popular method of resampling especially when some classes dominate others [4].

In the machine learning community, choosing the optimal features is a big deal that removes the irrelevant or less important features using wrapper methods or filter methods or embedded methods or learning-based methods.

Recently, the use of ensemble learning methods increases in the selection stage of the features. Extra Trees Classifiers outperform other peers in class categorization by selecting the optimal attributes besides computational efficiency [5].

The classifier performance speed is a design requirement during the planning of the systems in many applications especially those running in real time. For this reason, the extreme learning machine method is introduced as one of the fastest learning algorithms, surpassing dozens of learning techniques based on back-propagation [6].

The key metric for evaluating the classification issue is accuracy which is the number of correct predictions made from all predictions. In addition, the false alarm rate is a big deal when working on the classification to know how classifiers are powerful; i.e., they reduce the proportion of wrongly classified instances.

However, the classification accuracy alone is not sufficient information to make a proper decision. Therefore, in addition to the accuracy, care should be taken about ROC and PRC plots to avoid illogical results.

The research focuses on software machine learning-based network IDS using the abovementioned techniques from a classification problem viewpoint; it also sheds light on the accuracy, false alarm rate, ROC, and PRC.

The next parts of this research are organized as follows.  Section 2 presents summaries of related studies. Then, the proposed system is detailed in Section 3 as well as the used methodologies. Furthermore, results and discussion are given in Section 4. Finally, the conclusion is offered and further suggestions for future works are given in Section 5.

## 2. Literature Review

Studies varied over the selected dataset, i.e., UNSW-NB 15, depending on the type of attack or the protocol used, or the threat detection approach. So, some preferred to minimize the detection circuit to catch just one specified attack or perhaps two attacks at most. Others went toward discussing the problem relying on the transport layer protocol, i.e., TCP or UDP. Others did not do the multiclass classification, but they were satisfied with the binary classification.

This section focuses on state-of-the-art works connected to multiclass classifications on the selected dataset.

In [7], for each attack in the UNSW-NB15 dataset, they introduced a hybrid model for IDS based on a Genetic Algorithm (GA) and Support Vector Machine (SVM). They converted the features into chromosomes and selected the highest accuracy from them. Then, as a detection method, they proposed the Least Squares Support Vector Machine (LSSVM). The results were tested for accuracy, true positive rate, and false-positive rate.

In [8], a random forest (RF) was presented as a feature reduction method; they were interested in eight UNSW-NB 15 dataset attacks excluding “Fuzzers” attacks. They designed a stepwise architecture to detect attacks based on the random forest at each stage. The performance metrics for their study were false alarm rate (FAR) and the undetection rate (UND).

Deep learning methods have also been presented for the multiclass detection approach in anomaly-based detection. For example, the well-known Convolutional Neural Network (CNN) was used in [9], after converting features to 8 × 8 images to be entered into CNN layers. The classification accuracies were high for “Normal” and “Generic” traffic.

In [10], a combination of Artificial Bee Colony (ABC) and Artificial Fish Swarm (AFS) was declared for categorizing attacks. They split the dataset into subsets and used the Correlation-based Feature Selection (CFS) method to select the optimal attributes. After that, the CART technique was added to generate “If-Then” rules to be ready for the hybrid ABC-AFS. The performance was tested according to various values of the number of subsets.

Due to less complexity than other mixture models, a Beta Mixture Model (BMM) was performed as an anomaly-based detection technique in [11]. BMM uses a lower-upper interquartile threshold to distinguish between the normal and the abnormal profiles. They demonstrated their results in terms of detection rates for all attack classes and ROC curves.

Mixing multiple machine learning methods in studies is strongly recommended to exploit their strengths to improve the overall performance of IDS. For example, the study in [12] demonstrated that IDS can be achieved through a set of layers. The feature selection layer based on Extra Trees Classifiers for each threat was followed for detection by the extreme learning machine ensemble layer. Then, the outputs of the previous layer were collected with the softmax layer to make a soft decision for each attack. Results were limited to accuracy.

To ensure that the design of IDS models will make a good impression in production, multiple model experiments will be applied to many relevant datasets.

Thus, the study in [13] proposed distributed deep neural network (DNN) models with many hidden layers to monitor threats to the host level and the network level. Models have been tested in benchmark datasets. They released their framework “Scale-Hybrid-IDS-AlertNet” to detect cyberattacks in real time.

IDS architecture could be represented by levels according to the detection approaches such as [14] which explained this idea through a two-level design. The former was a model of binary classification based on a decision tree to detect benign and malignant flows. If malignant flows were predicted, the latter would start with a multiclass classification model based on a hybrid of Recursive Feature Elimination (RFE) and SMOTE to take precise decisions to categorize the abnormal flow.

In search of the high detection rates, the study in [15] illustrated their IDS by a combination of the Genetic Algorithm (GA) to delete irrelevant features and the Self-Organizing Map (SOM) classifier, optimized by GA's selected features.

In [16], they also used GA with random forest (RF) to select the optimum attributes, preceded by the Isolation Forest (iForest) for data sampling. A random forest (RF) classifier was reused to recognize the class type of attacks for a different goal. This suggestion produced high accuracies, high detection rates, and less false alarm rates.

IDS performance with reduction features outperforms others using all features. In [17], the optimal features were selected by applying Mutual Information with Linear Correlation Coefficient (MI-LCC), followed by the Support Vector Machine (SVM) classifier as a multiclass detection method.

Doing statistics of classes within a dataset helps scholars to design a robust IDS, particularly during the preprocessing stage, because machine learning models cannot be trained well whatever the models are at specific rates of classes.

Thus, in [18], the data were resampled using one-side selection (OSS) to decrease majority samples and SMOTE to increase minority samples. Then, the spatial features and the temporal ones were extracted by CNN and bidirectional long short-term memory (BiLSTM) respectively, which are the core of the classification stage by combining them.

In [19], they introduced their IDS for the cloud environment, using Chi-square as a feature selection method and deep reinforcement learning as a classification method. ROC curves showed accuracies, FPR, and TPR for each class.

Ensemble learning has been presented to enhance the detection rate in [20]. A long short-term memory (LSTM) algorithm, a homogeneous ensemble method, and a heterogeneous ensemble method based on multiple classifiers were implemented. The proposed models were tested on the selected dataset in two forms as a two-classed dataset and a multiclass dataset.

## 3. Our Proposed System

**Figure 1:**
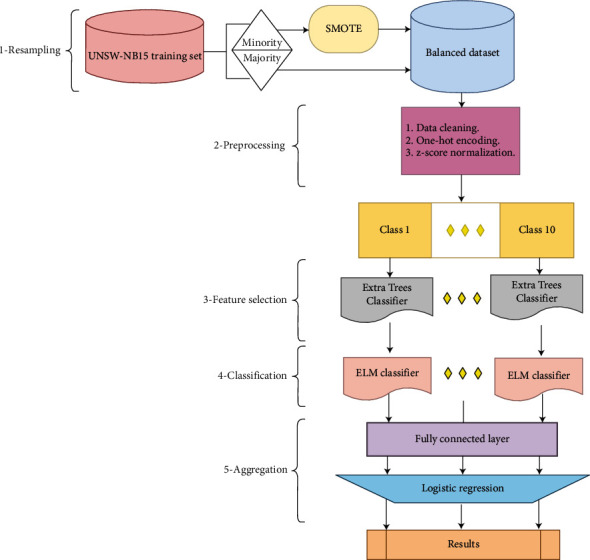
The proposed system.

After reviewing the future works related to the research topics, we noticed that the resampling techniques have improved the performance of the multiclass classification. As well, the methods of ensemble learning have done well for selecting the optimum features. Furthermore, the classification has implemented by machine learning rather than deep learning for more effective models with less complexity. As a consequence, our suggested IDS as shown in Figure 1 can be introduced, consisting of multiple stages.

### 3.1. Resampling

The unequal number of classes in a dataset badly affects the performance of the machine learning-based classifiers, especially when the majority of classes to the minority ones exceed 100 to 1, as many data scientists have stated. Because of the difficulty of creating a standard balanced dataset, preprocessing of the existing dataset should begin with decreasing the majority or increasing the minority or doing both. One of the simplest statistical techniques for dealing with the uneven categories in a dataset is SMOTE, which is applied to certain minority classes in the dataset selected.

#### 3.1.1. Synthetic Minority Oversampling Technique (SMOTE)

**Figure 2:**
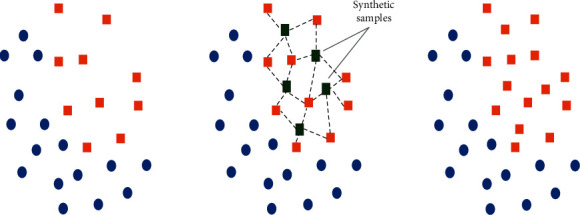
SMOTE work principle.

The basis for this method resides in the idea of oversampling the minority class by generating synthetic instances from its elements and keeping the majority number as is. The new samples are not only carbon copies of minority examples but are created by composing features from the minority instances and their closest neighbors in the feature space [4].  Figure 2 shows a simple way to oversample the minority cases (the orange squares) in the 2D feature space by drawing lines between them, and the synthetic minority instances reside the lines (the green squares). As well, the majority of cases (the circles) remain unchanged. As a result, the minority percentage only rose, and the classes are equal.

### 3.2. Preprocessing

Dealing with the raw data set examples requires some analysis and visualization of the values included. Some rows can be duplicated which causes overfitting problems. Some columns have dirty values such as spaces or nulls or various types of data.

To handle the above problems, the selected dataset should be preprocessed to make it free from any errors that affect the postprocessing process.

#### 3.2.1. Data Cleaning

Fixing the dataset flaws is an essential part which contains the following:

- Unification of textual values by changing the lower/upper/proper cases
- Treatment of nulls and spaces according to column attribute
- Convert the numbers stored as text type into a number type

#### 3.2.2. One-Hot Encoding

The nominal features should be converted to numerical values before fitting the machine learning models. One-hot encoding is a recommended approach to this.

It deals with categorical columns by creating new columns that are mapped to the number of distinct values inside. Each new column represents a single distinct category. It assigns ones matched to the category locations in the original column and the remainder are zeros.

#### 3.2.3. Z-Score Normalization

Each column has a different scale of its values after it gets numeric values in the dataset. There are some problems when fitting machine learning models because the features with large scales can dominate the others, making the results misleading. The goal of normalization is to equalize the importance of the features.

Z-score is one of the ways of the well-known normalization. Relation (1) expresses this strategy by subtracting its mean *μ* from every feature *x* and dividing the difference by its standard deviation *σ*:

(1) $$\begin{array}{c} z=\frac{x\;-\mu }{\sigma }. \end{array}$$

### 3.3. Feature Selection

This step is very important to design efficient machine learning models and to reduce the computational cost of high-dimensional feature space by selecting the most relevant features. Many techniques have been introduced for finding the optimal subset [21]:

- Wrapper methods: sequential selection algorithms and heuristic search algorithms
- Filter methods: correlation criteria and mutual information between features
- Embedded methods: MRMR (max-relevancy, min-redundancy) and L1 regularization
- Learning-based methods: some unsupervised/semisupervised/supervised/ensemble learning algorithms

No preferred methods are valid for any model of machine learning; some experiments should be done to find out which one achieves the best results based on the desired dataset or study problem.

Many strong recommendations claim that techniques of selection of features based on ensemble learning-based outperform other procedures especially Extra Trees Classifiers [5].

#### 3.3.1. Extremely Randomized Trees Classifier (Extra Trees Classifier)

One of the most common methods of tree-based ensemble machine learning. As claimed by [6], it gathers many randomized decision trees, without using bootstrapped samples. By using the entire training dataset, each decision tree has fitted in. It selects a split point randomly, based on a mathematical decision, to split tree nodes.

In the context of the suggested system, this algorithm has been exploited to capture the optimal features for each class in the dataset using the Gini Impurity criterion.

Gini Impurity measures the probability of incorrect classification of a particular feature when selected at random [22]. Its values range from 0 to 1; the lower the value is, the more important the relevant feature is.

### 3.4. Classification

It is a supervised learning approach that categorizes the examples of the dataset into groups by designing supervised learning models for this task. So, a labeled or categorized dataset is required to create models that map a subset of features to each class.

Based on the dataset, it can be binary classification when there are only two classes, or multiclass classification when the number of classes is greater than two, or multilabel classification when each instance is defined with multiple labels.

Hundreds of machine learning models can be declared as classifiers but the system goal, stability, complexity, scalability, and performance make researchers biased in favor of some algorithms over others. Therefore, the scope of application of the system must be determined before going into design.

For real-time applications, extreme learning machine methods with a low training time without iterative tuning, perfect generalization, and ease of implementation are strongly recommended.

In particular, these methods were introduced as candidates to apply them to the UNSW-NB15 dataset as mentioned in the survey [23].

#### 3.4.1. Extreme Learning Machines (ELMs)

According to [6], this algorithm is one of single-hidden-layer feedforward neural networks (SLFNs). It has been updated in many forms to improve their generalization ability and performance. However, the system selected has suggested the basic form applied to each class as a “One-Versus-All” binary classifier to make the processing easy and quick.

**Figure 3:**
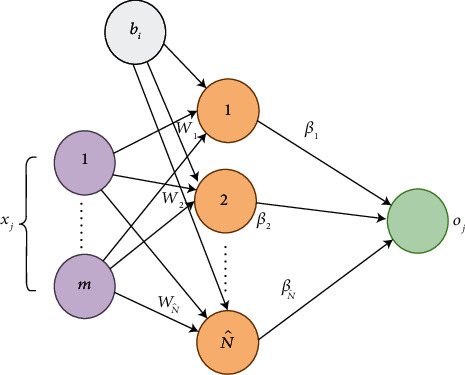
ELM network structure.

In general, it has only one hidden layer, with multiple neurons completely connected from one side to the input layer and from the other side to the output layer as declared in Figure 3.

From the previous figure, the ELM output applies the following mathematical relation:

(2) $$\begin{array}{c} \;\sum_{i=1}^{\hat{N}}\beta_{i}.\;g\left(w_{i}.x_{j}+b_{i}\right)=o_{j}\;\text{,}\;\text{for }j=1\text{ to }N, \end{array}$$

where we have the following:

- $\hat{N}$ is the number of hidden neurons
- *N* is the number of training instances
- *β*_*i*_ is the *i*th weight vector between the *i*th hidden neuron and the output layer
- *w*_*i*_ is the *i*th weight vector between the *i*th hidden neuron and the input layer
- *b*_*i*_ is the *i*th bias vector
- *g* is an activation function
- *x*_*j*_ is the *j*th input vector with *m* features
- *o*_*j*_ is the *j*th output sample

The error between the ELM output *o*_*j*_ and the actual target *t*_*j*_ in the perfect way should be zero as referred to

(3) $$\begin{array}{c} \sum_{j=1}^{N}\left\|o_{j}-t_{j}\right\|=0.\; \end{array}$$

As a result, formula (2) can be rewritten to become

(4) $$\begin{array}{c} \sum_{i=1}^{\hat{N}}\beta_{i}.\;g\left(w_{i}.x_{j}+b_{i}\right)=t_{j},\;\text{ for }j=1\text{ to }N. \end{array}$$

The matrix form of N equations in (4) is

(5) $$\begin{array}{c} H.\beta =T. \end{array}$$

For every *i*, *w*_*i*_ and *b*_*i*_ are randomly assigned without explicit intervention to calculate **H**; **T** is given in the dataset. The only thing to calculate is *β* as follows:

(6) $$\begin{array}{c} \hat{\beta }=H^{†}T, \end{array}$$

where **H**^†^=(**H**^**T**^**H**)^−1^ **H**^**T**^ is the Moore–Penrose generalized inverse of matrix **H**. $\hat{\beta }$ is proven to be the optimal solution for the least-squares error:

(7) $$\begin{array}{c} \left\|H\hat{\beta }-T\right\|=\min_{\beta }\left\|H\beta -T\right\|. \end{array}$$

From the complexity standpoint, thanks to the simplicity of its structure, ELM significantly reduces computational burdens.

### 3.5. Aggregation

After creating classification models for each class, their outputs are simultaneously collected in order to design the aggregated model. This architecture makes the proposed system scalable to add any new classes.

The aggregated model is a fully connected layer which is followed by a layer of logistic regression. The fully connected layer is very important for capturing all combinations of ELM classifier outputs to improve the classification.

In order to be able to distinguish between all classes, we were interested in the multinomial form of the logistic regression layer. The multinomial logistic regression uses maximum likelihood estimation using Newton's method [24].

The softmax function was represented as an activation function for the logistic regression layer with ten neurons such as the length of the input vector to make soft decisions at the output.

The softmax function is defined as follows:

(8) $$\begin{array}{c} f{\left(z\right)}_{i}=\frac{e^{z_{i}}}{\sum_{k=1}^{n}e^{z_{k}}},\;\text{for }i=1\text{ to }n, \end{array}$$

where *z*_*i*_ is the input vector of the neural network with *n* neurons. This stage makes the system dealing with the IDS a problem of multiclass classification.

In order to improve overall performance, the Adam optimization method was chosen to leverage the simplicity and computational efficiency [25].

Working with information content (entropy) is very intuitive when handling probabilities; sparse categorical cross-entropy has been used as a cost function for multiclass classification tasks with the softmax layer [26]. In this way, the cross-entropy *H* between two probability distributions *p*, *q* is

(9) $$\begin{array}{c} H\left(p,q\right)=-\sum_{i}p_{i}.\;\log\left(q_{i}\right). \end{array}$$

Along these lines, the proposed system has offered multiple stages defined by algorithms to be as flexible, fast, and simple as possible.

## 4. Experimental Setup and Results

Our proposed system was developed using Python language. It was run on the 8^th^ generation intel core i7 processor and an 8 GB RAM.

Some details about the UNSW-NB15 dataset should be provided before diving into the results. Due to its advantages over old standard datasets, this dataset is chosen. KDD98, KDDCUP99, and NSLKDD datasets are suffering from the lack of modern cyberattack types, inadequate normal traffic, and the unequal distribution of classes in training and testing sets. The UNSW-NB15 has been presented as a benchmark dataset specialized in IDS design [27] to address these problems.

### 4.1. UNSW-NB15 Dataset

According to [28], the Cyber Range Lab of the Australian Centre for Cyber Security (ACCS) at UNSW in Canberra presented the new UNSW-NB15 dataset, considering the limitations of the old existing dataset. IXIA PerfectStorm tool has been used to create a combination of recent malicious and benign behaviors of network traffic.

The dataset consists of nine types of modern cyberattacks labeled by Analysis, Backdoors, DoS, Exploits, Fuzzers, Generic, Reconnaissance, Shellcode, and Worms in addition to the normal packets, named as Normal, which were captured using the Tcpdump tool.

The packets within the dataset are defined by 49 different features provided by the Argus, Bro-IDS tools, and twelve additional algorithms.

The most used UNSW_NB15_training-set.csv including 175,341 records and UNSW_NB15_testing-set.csv including 82,332 records are partial datasets and publicly available to help researchers develop IDS in training and testing issues, respectively.  Table 1 shows the samples for each class and their percentage.

### 4.2. Performance Metrics

The best ways to illustrate the classification results while applying supervised learning models are accuracy, precision, recall, F1-score, false alarm rate, ROC, and PRC.

#### 4.2.1. Confusion Matrix

It collects the results of properly and incorrectly classified samples for each class, either for binary classifiers as shown in Table 2 or for multiclass classifiers.

In Table 2, we have the following:

- TP: attack samples are correctly classified as attack samples
- FP: normal samples are classified as attack samples
- TN: normal samples are correctly classified as normal samples
- FN: attack samples are classified as normal samples

From the confusion matrix, some equations can be defined [29]:

(10) $$\begin{array}{c} \text{accuracy}=\frac{\text{TP}+\text{TN}}{\text{TP}+\text{FP}+\text{TN}+\text{FN}},\\ \text{precision}=\frac{\text{TP}}{\text{TP}+\text{FP}},\\ \text{recall}=\frac{\text{TP}}{\text{TP}+\text{FN}}=\text{sensitivity}=\text{TPR, }\\ \text{specificity}=\frac{\text{TN}}{\text{FP}+\text{TN}},\\ F1-\text{measure}=\frac{2*\text{precision}*\text{recall}}{\text{precision}+\text{recall}}.\; \end{array}$$

False alarm rate (FAR) is one of the important measures that focus on misclassified ratios, which is the average between the ratio of misclassified samples over all normal samples called false positive rate (*FPR*) and the ratio of misclassified samples over all attack samples called false-negative rate (*FNR*) [28]:

(12) $$\begin{array}{c} \text{FPR}=1-\text{specificity}=\frac{\text{FP}}{\text{FP}+\text{TN}},\\ \text{FNR}=\frac{\text{FN}}{\text{TP}+\text{FN}},\\ \text{FAR}=\frac{\text{FPR}+\text{FNR}}{2}. \end{array}$$

#### 4.2.2. Receiver Operating Characteristics (ROC)

The ROC curve is a 2D graphical plot with a true positive ratio (TPR) on the *y*-axis against a false positive rate (FPR) on the *x*-axis [30]. To show how classifiers distinguish between two classes, it draws lines between thresholds that are determined when making decisions in binary classification. One common measure with the ROC curve is the area under the curve (AUC) with values between 0 and 1. Higher AUC (more than 0.5) measures how well-trained classifiers are by allocating higher probability for correct predictions and lower probability for incorrect ones. A badly trained classifier has a diagonal line ROC curve with AUC close to 0.5.

#### 4.2.3. Precision-Recall Curve (PRC)

PRC is an alternative metric for the proper evaluation of binary classifiers for an imbalanced dataset. Like its name, it is a visual plot showing how precision on the *y*-axis is linked to recall on the *x*-axis [31].

For each decision threshold to construct the curve of PRC, multiple pair points of recall and precision are defined, respectively. Also, AUC is used with PRC in the same meaning with ROC curves.

### 4.3. Results

As shown in Figure 1, the implementation of the proposed system consists of several phases that are applied to the training set. The training set is divided into 80% for training and 20% for validation. The results are shown only for the testing set.

#### 4.3.1. Resampling

SMOTE is used to oversample the minority classes whose percentage in the training set is less than 2%, which are Analysis, Backdoors, Shellcode, and Worms. The other classes are kept without any resampling.

#### 4.3.2. Preprocessing

*Data Cleaning*. Some instances whose values are spaces and “-” are dropped. Some numeric values which are stored as text types are converted into number types. Because some types of attacks exist for the same attack name in different syntaxes such as the upper and lower cases, they are unified to the same format. Null values are replaced by the median of the feature column.

*One-Hot Encoding*. One-hot encoding encodes the three nominal attributes (*proto*, *state,* and *service*) to get new columns filled with ones and zeros.

*Z-Score Normalization*. After numeric columns are obtained, a z-score is implemented to normalize the attributes scales for every single column.

#### 4.3.3. Feature Selection

**Figure 4:**
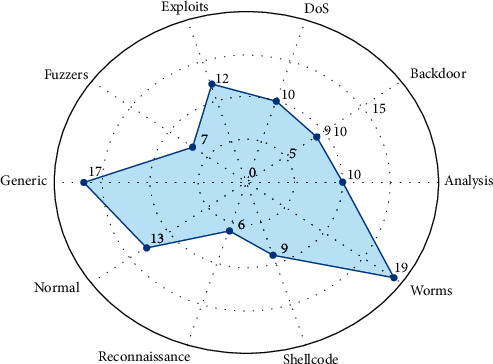
Number of extracted features from Extra Trees Classifiers by classes. For classification purposes, each Extra Trees Classifier produces a subset of features that are fed to the appropriate ELM classifier.

Gini Impurity criterion is used as a decision-maker for Extra Trees Classifier to extract the optimum features for every class in the dataset. After testing multiple Gini Impurity values, the best value is 0.02 which eliminates the number of features, as shown in Figure 4, to the minimum with no overall performance degradation at all.

#### 4.3.4. Classification

**Figure 5:**
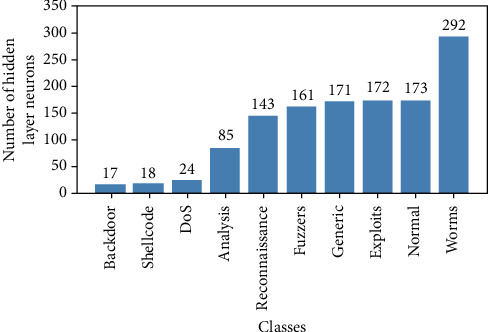
Number of hidden layer neurons of ELM classifiers by classes.

For each ELM classifier, the chosen activation function is “ReLU”, and the number of neurons is iteratively tuned to achieve the best results. In the graph shown in Figure 5, the final numbers for the single hidden layer of each ELM classifier are obtained.

**Figure 6:**
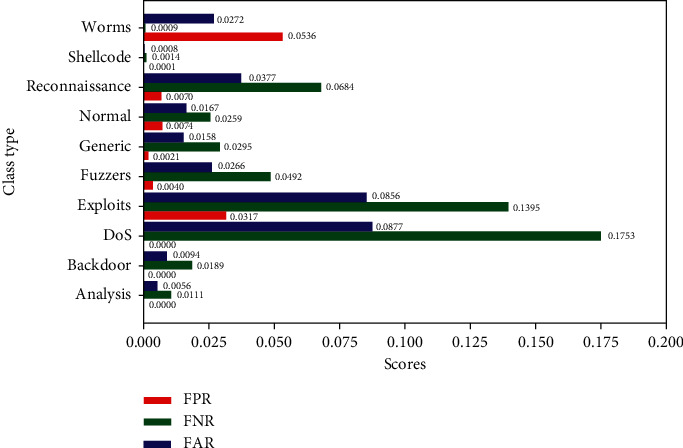
FPR, FNR, and FAR of ELM classifiers by classes.

**Figure 7:**
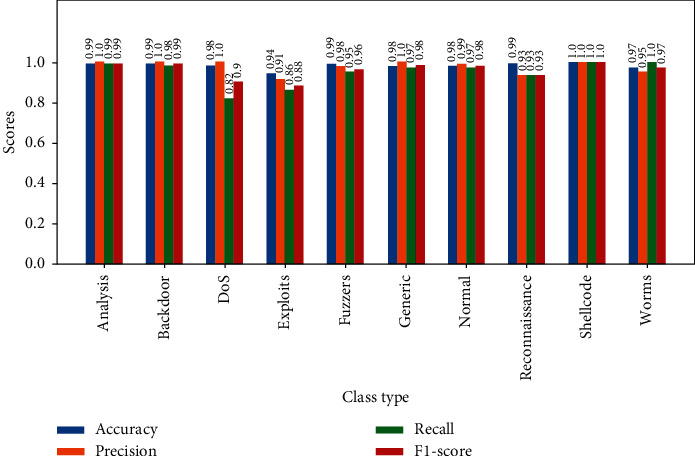
Classification metrics of ELM classifiers by classes.

**Figure 8:**
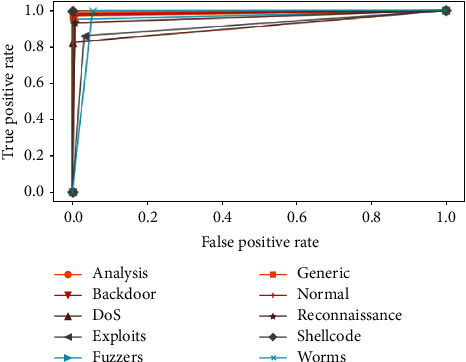
ROC curves of ELM classifiers by classes.

**Figure 9:**
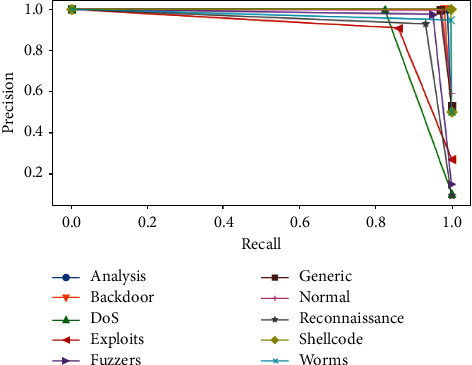
Precision-Recall curves of ELM classifiers by classes.

**Figure 10:**
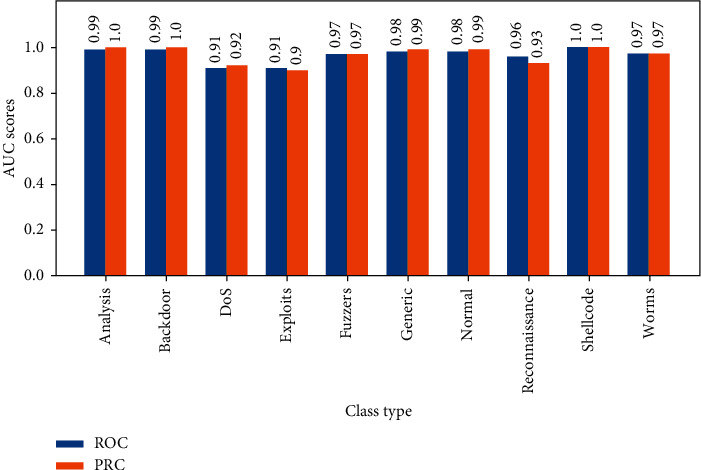
AUC for ROC and PRC of ELM classifiers by classes.

In the graphs shown in Figures 6–10, the classification results of the ELM classifiers are collected by applying them to the testing set for each category.  Figure 6 shows the per class FPR, FNR, and FAR.  Figure 7 illustrates the accuracy, precision, recall, and F1-score.

ROC curves are drawn in Figure 8 for each binary classifier, and Precision-Recall curves are plotted in Figure 9. AUC for ROC and Precision-Recall curves are grouped as shown in Figure 10.

#### 4.3.5. Aggregation

The classification results of the ELM classifiers are gathered to feed a logistic regression layer with a softmax as an activation function to make soft decisions for each class. Also, as a loss function, the sparse categorical cross-entropy is used, and Adam is chosen as the optimizer. The overall accuracy is ultimately 0.9843.

### 4.4. Comparison with Related Works

After numeric results have been shown, our suggested system can be compared with other related studies in order to realize the importance of performance improvement of the multiclass classification that our system is presenting.

The comparison metrics available are the accuracy, TPR, FPR, and F1-score as outlined in Tables 3, 4, 5, and 6, respectively.

### 4.5. Discussion

The results offered for comparison with the proposed system in [8, 12–19] are obtained using the partial datasets which are shown in Table 1. However, other results in [7, 9–11] are obtained using the full datasets, which are 2,540,044 records including training sets and testing sets [28].

The suggested IDS, as indicated throughout this paper, is a combination of SMOTE, Extra Trees Classifiers. and ELM classifiers. SMOTE makes it possible to classify minority classes, rather than ignoring them as in [7, 13, 16]. In addition, Extra Trees Classifiers are selected to obtain the minimum number of features, as shown in Figure 4, as compared to [7, 17].

Some studies are more interested in recall rather than in other scores, for example, in [10, 11, 13, 14, 15, 16]. It makes sense that recall, as the ratio of the correctly classified attacks over all attack samples, is the important metric of anomaly detection problems. In these issues, we are focusing more on attack samples than normal ones because the damage to incorrectly classified attacks from attack samples is greater than when the samples are normal.

Some related studies focus on accuracy only, such as [9, 12]. However, the accuracy of the classification does not provide enough information about the robustness of machine learning models. Other metrics such as precision, recall, F1-score, and false alarm rate must be taken into consideration during the design of these models.

For example, assuming that a dataset contains several packets, 99% of packets are labeled as normal whereas only 1% are labeled as abnormal. Assume that a model is trained somehow to classify all packets as normal. So, the accuracy will be 99%. Although the classification accuracy is high, logically, the result is disappointing as it will not be able to detect any attacks.

As the number of features extracted from Extra Trees increases as shown in Figures 4 and 5, the number of hidden layer nodes of ELMs increases as another notable issue.

Figure 6 shows that the FNR values for most classes are greater than the FPR values because the classifiers are trained by a large number of samples that are not related to a single attack relative to the small number of the attack samples. That makes the false decisions about samples which are not related to this attack smaller than the false decisions about the attack samples.

Our proposed system is designed to make the false alarm rate as low as possible in general without lowering the accuracy, recall, precision, and F1-score; a trade-off is needed.

In Figure 10, we observe that ROC and PRC AUCs are nearly equal for each class. This shows the strong relationship between each of the two curves related to a single class. The two AUCs are generally different, especially when the classes are very imbalanced. However, thanks to SMOTE, the two AUCs are close enough through balancing the classes.

There are some noteworthy observations from the results; on the same dataset, the proposed system has outperformed other classification algorithms as explained numerically in Tables 3, 4, 5, and 6, in particular, the lowest false positive rates and the highest accuracy, detection rates, and F1-scores.

## 5. Conclusion

As a result, our proposed system has presented a multiclass classifier for all existing categories in the standard dataset with soft decisions. We have shown that it implicitly introduces a binary classifier to detect normal and attack packets because one of the ELM classifiers is for the normal class which is defined by the “One-Versus-All” methodology.

It contains multiple stages defined by algorithms such as SMOTE, Extra Trees Classifiers, and ELM that were chosen to be as flexible, fast, and simple as possible. So, it can easily run on low-performance hardware. Finally, the results are displayed in terms of accuracy, precision, recall, F1-score, false alarm rate, ROC, and Precision-Recall curves to confirm the quality of classifiers.

For future work, the parallel approach makes the system smoothly scalable for any new attacks as new binary classifiers. The proposed system can also be preceded by an unsupervised learning stage to detect normal and abnormal traffic without labels. If the abnormal behavior sounds like one of the classes that existed, it is categorized more specifically within the system.

## Figures and Tables

**Table 1:** A part of UNSW-NB15 dataset distribution.

Class type	Training samples	Training samples percentage	Testing samples	Testing samples percentage
Normal	56000	31.94	37000	44.94
Analysis	2000	1.14	677	0.82
Backdoors	1746	1.00	583	0.71
DoS	12264	6.99	4089	4.97
Exploits	33393	19.05	11132	13.52
Fuzzers	18184	10.37	6062	7.36
Generic	40000	22.81	18871	22.92
Reconnaissance	10491	5.98	3496	4.25
Shellcode	1133	0.65	378	0.46
Worms	130	0.07	44	0.05
**Total**	**175341**	**100**	**82332**	**100**

**Table 2:** A confusion matrix for binary classification.

Actual/predicted	Attack	Normal
Attack	True positive (TP)	False negative (FN)
Normal	False positive (FP)	True negative (TN)

**Table 3:** Comparing the proposed system with related works on accuracy.

Method							
Class	GV-SVM [7]	RF [8]	CNN [9]	ELM [12]	LCC-MI-SVM-FS [17]	RF [18]	The proposed system
Normal	97.45	99.50	**99.7**	91.26	75.8	—	98.16
Analysis	—	2.00	0	98.96	99.1	84.67	**99.44**
Backdoors	—	5.00	0	99.11	**99.2**	83.53	99.06
DoS	91.24	20.00	0	94.75	94.9	92.12	**98.14**
Exploits	79.19	**99.50**	61.8	89.13	84.2	79.21	93.91
Fuzzers	96.39	—	6.8	91.30	91.6	93.43	**98.92**
Generic	97.51	97.00	97.7	98.16	91.5	96.37	**98.34**
Reconnaissance	91.51	86.00	0	94.60	95.7	89.45	**98.74**
Shellcode	99.45	80.00	0	99.40	99.5	92.79	**99.92**
Worms	—	70.00	0	**99.92**	99.9	65.31	97.28

**Table 4:** Comparing the proposed system with related works on TPR.

Method								
Class	GV-SVM [7]	CFS-ABC-AFS [10]	BMM-ADS [11]	5-DNN [13]	RFE-SMOTE [14]	SOM-GA [15]	DO-IDS [16]	The proposed system
Normal	98.47	92.8	93.4	92.8	**100**	88.3	96.7	97.41
Analysis	—	80.11	83.4	0	18	58.8	6.1	**98.89**
Backdoors	—	63.4	63.8	34.4	11	64.7	40.3	**98.11**
DoS	91.22	83.3	89.6	**97.7**	32	66.9	46.1	82.47
Exploits	67.31	63.7	79.4	1.3	82	79.1	66.3	**86.05**
Fuzzers	94.39	60.3	52.8	0	89	57.5	38.1	**95.08**
Generic	96.69	87.3	86.3	57.1	99	89.1	96.9	**97.05**
Reconnaissance	87.15	49.3	55.6	1.8	76	78.1	82.0	**93.16**
Shellcode	**100**	70.9	48.7	0	88	55.0	78.0	99.86
Worms	—	55.3	47.8	0	16	65.9	79.5	**99.91**

**Table 5:** Comparing the proposed system with related works on FPR.

Method							
Class	GV-SVM [7]	RF [8]	5-DNN [13]	DO-IDS [16]	LCC-MI-SVM-FS [17]	RF [19]	The proposed system
Normal	0.04	—	0.285	0.033	0.383	—	**0.0074**
Analysis	—	0.0056	0	0.39	0	0.016	**0**
Backdoors	—	0.0005	0.013	0.597	0	0.018	**0**
DoS	0.08	0.002	0	0.539	0.0018	0.01	**0**
Exploits	0.06	0.014	0	0.337	0.1081	**0.009**	0.0317
Fuzzers	0.01	—	0	0.619	0.0169	0.012	0.0040
Generic	0.01	**0.00091**	0.166	0.031	0.0234	0.008	0.0021
Reconnaissance	0.02	0.007	0.008	0.180	**0**	0.014	0.0070
Shellcode	0.09	0.006	0	0.220	0	0.016	0.0001
Worms	—	0	0	0.205	0	0.019	0.0536

**Table 6:** Comparing the proposed system with related works on F1-score.

Method				
Class	RFE-SMOTE [14]	DO-IDS [16]	CNN-BiLSTM [18]	The proposed system
Normal	**100**	93.0	84.99	98.43
Analysis	28	5.3	9.69	**99.44**
Backdoors	18	21.9	8.97	**99.05**
DoS	34	39.9	29.55	**90.39**
Exploits	72	70.8	67.89	**88.45**
Fuzzers	91	54.2	37.47	**96.34**
Generic	**99**	98.3	98.85	98.41
Reconnaissance	84	85.3	62.54	**93.11**
Shellcode	87	48.6	30.95	**99.92**
Worms	25	78.7	10.75	**97.34**

## Data Availability

The UNSW-NB15 data sets are available at https://www.unsw.adfa.edu.au/unsw-canberra-cyber/cybersecurity/ADFA-NB15-Datasets/.
